# Functional polymorphisms of the mineralocorticoid receptor gene *NR3C2* are associated with diminished memory decline: Results from a longitudinal general‐population study

**DOI:** 10.1002/mgg3.1345

**Published:** 2020-06-18

**Authors:** Jan Terock, Sandra Van der Auwera, Deborah Janowitz, Katharina Wittfeld, Alexander Teumer, Hans J. Grabe

**Affiliations:** ^1^ Department of Psychiatry and Psychotherapy University Medicine Greifswald Greifswald Germany; ^2^ Department of Psychiatry and Psychotherapy Helios Hanseklinikum Stralsund Stralsund Germany; ^3^ German Center for Neurodegenerative Diseases (DZNE) Greifswald Germany; ^4^ Institute for Community Medicine University Medicine Greifswald Greifswald Germany

**Keywords:** cognitive decline, mineralocorticoid receptor, NR3C2 gene, rs2070951, rs5522

## Abstract

**Background:**

The mineralocorticoid receptor (MR) in the brain has a key role in the regulation of the central stress response and is associated with memory performance. We investigated whether the genetic polymorphisms rs5522 and rs2070951 of *NR3C2* showed main and interactive effects with childhood trauma on memory decline.

**Methods:**

Declarative memory was longitudinally assessed in 1,318 participants from the community‐dwelling Study of Health in Pomerania using the Verbal Learning and Memory Test (VLMT). In a subsample of 377 participants aged 60 and older, the Mini‐Mental Status Examination (MMSE) was additionally applied. Mean follow‐up time for the VLMT and MMSE were 6.4 and 10.7 years, respectively.

**Results:**

Homozygous carriers of the G allele of rs2070951 (*p* < .01) and of the A allele of rs5522 (*p* < .001) showed higher immediate recall of words as compared to carriers of C allele (rs2070951) or the G allele (rs5522). The CG haplotype was associated with decreased recall (*p* < .001). Likewise, in the subsample of older patients, the AA genotype of rs5522 was associated with higher MMSE scores (*p* < .05). CG haplotypes showed significantly reduced MMSE scores in comparison to the reference haplotype (*β* = −0.60; *p* < .01).

**Conclusions:**

Our results indicate that the GG genotype of rs2070951 as well as the AA genotype of rs5522 are associated with diminished memory decline.

## Graphical abstract

The mineralocorticoidreceptor has a central role in the brains stress response. In this longitudinal general population study, we investigated whether rs5522 and rs2070951 of the *MR* gene are associated with memory performance. Using two different measures for memory performance, we found that these single nucleotide polymorphisms were associated with diminished declarative memory decline.

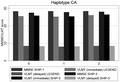

## 1 INTRODUCTION

Exposure to severe or lasting stress has consistently been reported to be associated with impaired memory performance and faster cognitive decline (Schwabe, Joëls, Roozendaal, Wolf, & Oitzl, 2012). For example, in their large population‐based study, Aggarwal et al. (2014) reported that chronic perceived stress predicted a faster rate of cognitive decline in subjects aged 65 or older. Peavy et al. (2009) showed in their longitudinal study that event‐based stress was associated with faster cognitive decline in subjects with mild cognitive impairment.

Stress induces an activation of different neuroendocrine systems including the hypothalamic–pituitary–adrenal (HPA) axis, leading to a release of cortisol, the primary factor of physiological and behavioral stress adaption (Miller, Chen, & Zhou, 2007). There is converging evidence showing that elevated levels of circulating cortisol are associated with impaired cognitive capacities and particularly the declarative memory (Lee et al., 2007; Lupien, Maheu, Tu, Fiocco, & Schramek, 2007). Moreover, excessive cortisol levels were repeatedly found to be associated with cognitive decline and memory loss: for example, Karlamangla, Singer, Chodosh, McEwen, and Seeman (2005) found that elevated urinary cortisol levels predicted incident cognitive impairment over a 7 year follow‐up. Greendale, Kritz‐Silverstein, Seeman, and Barrett‐Connor (2000) reported that higher basal cortisol levels predicted verbal memory loss in postmenopausal women. In another longitudinal study, declarative memory performance decreased over a 3 year period in subjects with higher cortisol levels (Li et al., 2006).

Cortisol exerts its effects in the brain by binding to two different intracellular receptors, the glucocorticoid receptor (GR) and the mineralocorticoid receptor (MR). GR shows a relatively low affinity to cortisol and are progressively occupied with increasing cortisol levels under conditions of acute stress (Harris, Holmes, De Kloet, Chapman, & Seckl, 2013). In contrast, the intracellular MR has a 10‐fold higher affinity to cortisol leading to a high occupancy of cortisol even under baseline conditions (De Kloet et al., 2016). These two hippocampal steroid receptors are considered to exert antagonistic actions with the MR mediating the tonic influences of basal cortisol levels and the GR facilitating negative feedback actions in response to increasing cortisol levels in order to maintain steroidal homeostasis (De Kloet & Reul, 1987; Harris et al., 2013). More specifically, Joëls (2006) demonstrated that MR and GR in the CA1 area of the hippocampus, a brain region with particular relevance for learning and memory, facilitate a U‐shaped dose‐response relationship with cortisol levels such that low levels sufficient to activate MR lead to efficient long‐term potentiation while high cortisol levels result in diminished long‐term potentiation via GR activation. The complementary and partly opposing effects of the MR and the GR are associated with specific effects on neuronal function (Joels, Karst, DeRijk, & de Kloet, 2008), behavior (Oitz, van Haarst, & de Kloet, 1997), and emotions (Brinks, Berger, Gass, de Kloet, & Oitzl, 2009). In addition to the intracellular MR, membrane‐located MRs were identified which have a significantly lower cortisol affinity compared to the intracellular receptor and which mediate rapid effects of cortisol particularly on hippocampal excitability (Karst, Berger, Erdmann, Schütz, & Joëls, 2010). Given the lower cortisol affinity, these membrane‐located MR may be important in modulating the neuroendocrine response to acute stress.

Expression and activity of the MR are influenced by different functional single nucleotide polymorphisms (SNP) of the MR encoding gene *NR3C2*. Specifically, the SNPs MR‐2C/G (rs2070951) and MRI180V (rs5522) were found to modulate MR activity by altering expression, transactivation, and activity (Van Leeuwen et al., 2011; for review ter Heegde, De Rijk, & Vinkers, 2015). Their results revealed a low linkage disequilibrium (*r* ^2^ = .11) of the corresponding SNPs and the occurrence of three common haplotypes based on these SNPs (Van Leeuwen et al., 2011). In vitro studies showed that the C allele of rs2070951 is linked to increased expression of MR, while the valine (G) allele of rs5522 is associated with a loss of MR function as compared to the isoleucine allele (A) (DeRijk et al., 2006; Van Leeuwen et al., 2011). The SNPs form three haplotypes being frequently found in general‐population samples, while the remaining haplotype (GG) hardly occurs (DeRijk, van Leeuwen, Klok, & Zitman, 2008).These haplotypes were reported to impact on MR functionality with the GA haplotype (“1”) showing the highest frequency in the general population (approx. 50%) and a lower expression, transactivation, and activity as compared to the other haplotypes (haplotype 2: CA; haplotype 3: CG) in response to cortisol stimulation (Klok et al., 2011; Klok et al., 2011). However, under basal, nonstimulated conditions, haplotype 3 showed the lowest functionality in in vitro testing (Klok et al., 2011; Klok et al., 2011).

While the GR is expressed ubiquitously in the brain, MR density is highest in limbic regions such as the hippocampus and the amygdala (Seckl, Dickson, Yates, & Fink, 1991), brain regions with a critical role in learning and memory functioning (Eichenbaum, 2000). Moreover, the hippocampus and related areas are particularly sensitive to the detrimental effects of prolonged cortisol elevations, which may contribute to explain the link between chronic stress and memory impairments. In fact, results from human and animal studies showed that chronically elevated levels of corticosteroids are associated with hippocampal damage and decreased performance in hippocampus‐dependent memory tasks (Issa, Rowe, Gauthier, & Meaney, 1990; Lupien et al., 1998; Ohl, Michaelis, Vollmann‐Honsdorf, Kirschbaum, & Fuchs, 2000). For example, it was found that chronically elevated levels of cortisol in depressed subjects or individuals with Cushing's disease were associated with hippocampal atrophy and impaired verbal declarative memory performance (O’Brien, Lloyd, McKeith, Gholkar, & Ferrier, 2004; Starkman, Gebarski, Berent, & Schteingart, 1992).

Considering these findings, MR functionality as determined by common functional polymorphisms may moderate the effects of chronic stress and elevated cortisol levels on brain damage and memory decline. Childhood trauma is a chronic stress factor which is associated with lasting neuroendocrine disturbances (Heim, Newport, Mletzko, Miller, & Nemeroff, 2008; Terock, Hannemann, Janowitz, Freyberger, et al., 2019; Terock, Hannemann, Janowitz, Van der Auwera, et al., 2019). Subjects with a history of childhood trauma were found to show blunted (Carpenter et al., 2007; Goldman‐Mellor, Hamer, & Steptoe, 2012) or increased activity (Heim et al., 2000) in response to acute stress and it has been suggested that the neuroendocrine dysregulations found in traumatized subjects may contribute to explain the association of childhood trauma with neuropsychological deficits (Kessler et al., 2010). Moreover, childhood trauma was repeatedly found to interact with genes involved in the HPA‐axis regulation in their effects on different psychopathologies (Binder et al., 2008; Terock et al., 2019). In a recent study of our working group, we found that childhood trauma and particularly childhood emotional neglect was significantly associated with impaired verbal declarative memory performance (Terock et al., 2019). Finally, childhood adversities were found to be associated with structural alterations of brain regions relevant to memory performance including the hippocampus and the prefrontal cortex (for review: [Teicher, Samson, Anderson, & Ohashi, 2016]).

In addition, different experimental studies were concerned with the role of the MR for different memory domains: For example, Rimmele, Besedovsky, Lange, and Born (2013) reported that administration of spironolactone, a selective MR antagonist compared to placebo impaired free recall of both texts and pictures particularly for emotional information. In contrast, Otte et al. (2007) found impaired visuospatial memory, but not verbal declarative memory after administration of spironolactone in healthy young men. Cornelisse et al. investigated measures of memory performance in relation to spironolactone administration and exposure to acute psychosocial stress. The authors found no general effect of spironolactone treatment alone on verbal memory and an even enhancing effect of psychosocial stress on delayed word recall after 24 hr in participants who received spironolactone (Cornelisse, Joëls, & Smeets, 2011). On the other hand, there is evidence that not only blockade, but also stimulation of the MR impacts on memory formation and retrieval: Hinkelmann et al. (2015) reported that administration of fludrocortisone, a MR agonist, resulted in improved performance of different memory domains. Regarding verbal learning, their results showed positive effect on trend‐level of fludrocortisone treatment for early recall of a word list and no effect for delayed recall of words. In another placebo‐controlled study investigating autobiographical memory in healthy subjects as well as patients with major depressive disorder (MDD) or borderline personality disorder, MR stimulation with fludrocortisone did not result in altered memory performance (Fleischer et al., 2015).

Moreover, there is evidence from animal and human studies showing that stress and emotional arousal modify the engagement of different memory systems such that dorsal‐striatum dependent habitual memory functions become more active in relation to hippocampus‐dependent declarative memory under conditions of acute stress (Packard & Wingard, 2004; Schwabe et al., 2007; Schwabe, Schächinger, de Kloet, & Oitzl, 2009) and that this shift is mediated by stress induced MR stimulation. For example, Schwabe, Tegenthoff, Höffken, and Wolf (2013) showed that an acute stress task was associated with reduced explicit task knowledge and a relative increase in procedural learning, as well as altered coupling of the amygdala with hippocampal and dorsal‐striatal structures in humans. These effects could be prevented by MR blockade prior to the stress test. In addition, Wirz, Reuter, Wacker, Felten, and Schwabe (2017) found that the effect of MR activation on the shift in learning strategies and memory engagement was modulated by the *NR3C2* polymorphisms.

Results from preceding animal studies indicated that MR expression modulates the effects of chronic stress on brain structures and function (ter Heegde et al., 2015). However, results from human studies are few and revealed mixed results: Kuningas et al. (2007) prospectively investigated the effects of cortisol and polymorphisms in the GR and MR genes on global cognitive functioning in subjects aged 85 years and older. While cortisol levels were associated with reduced global cognitive functioning at baseline and at follow‐up, none of the tested genetic polymorphisms emerged as a predictor for cognitive functioning in old age. Gerritsen, Comijs, Deeg, Penninx, and Geerlings (2011) performed a study testing 30 genetic polymorphisms involved in the regulation of the HPA‐axis in their effects on different cognitive, endocrine, and brain structural outcomes. While the authors found no main effects of the *NR3C2* on any endocrine or brain morphological outcome, they reported that the strongest interactive effect emerged for the interaction of childhood maltreatment and a functional polymorphism in the *NR3C2* on smaller hippocampal volume. In all, results from previous studies suggest that the MR plays an important role in moderating the effects of increased cortisol levels and prolonged stress on altered memory functioning and memory decline. Moreover, given the associations of childhood trauma with both, HPA‐axis activity as well as memory impairments, it could be speculated that differences in MR functioning may contribute to explain the association of childhood trauma with reduced memory performance.

Based on these findings, we sought to investigate the main and putative interaction effects of childhood trauma and the SNPs rs2070951 and rs5522, as well as the combined haplotypes and diplotypes on verbal declarative memory performance in a general population sample and using a longitudinal study design. As an internal validation, we also tested whether these putative effects also apply to global cognitive functioning in a subsample of older patients.

## 2 MATERIALS AND METHODS

### 2.1 Sample

We analyzed data from the Study of Health in Pomerania (SHIP) (Völzke et al., 2011) comprising adult German residents in northeastern Germany. From the total population of West Pomerania comprising 213,057 inhabitants in 1996, a two‐stage stratified cluster sample of adults aged 20–79 years was drawn. The net sample (without migrated or deceased persons) comprised 6,265 eligible subjects. 4,308 Caucasian subjects participated at baseline SHIP‐0 (1997–2001). To date, three regular follow‐ups have been carried out (SHIP‐1 with *n* = 3,300 from 2002 to 2006; SHIP‐2 with *n* = 2,333 from 2008 to 2012 and SHIP‐3 with *n* = 1,718 from 2014 to 2016). In parallel to SHIP‐2, detailed assessments of life events and mental disorders were conducted within the SHIP‐LEGEND study (Life Events and Gene–Environment Interaction in Depression) from 2007 to 2010 including *n* = 2,400 participants from the SHIP‐0 baseline sample. Each SHIP survey included a computer‐assisted personal interview and a large range of standardized medical examinations. Socio‐demographic characteristics as well as information on behavioral risk factors and medical history were collected. Education measured as the number of schooling years was divided into <10/=10/>10 years.

From the analyses, we excluded those participants with missing information in any of the exposure, outcome, or confounding variables, as well as participants without longitudinal information on memory performance. This yielded a study population of *n* = 1,318 for the Verbal Learning and Memory Test (VLMT) analyses and *n* = 377 for Mini‐Mental Status Examination (MMSE) (Folstein, Robins, & Helzer, 1983).

The investigations in SHIP were carried out in accordance with the Declaration of Helsinki, including written informed consent of all participants. The survey and study methods were approved by the institutional review boards of the University of Greifswald.

### 2.2 Instruments

Amongst others, subjects of SHIP‐LEGEND and SHIP‐3 were administered the Auditory VLMT, a German adaption of the widely used Rey Auditory Verbal Learning Test (Helmstaedter & Durwen, 1990). In addition, the subjects of SHIP‐LEGEND completed the childhood trauma questionnaire (CTQ) (Bernstein & Fink, 1998; Wingenfeld et al., 2010). Subjects of SHIP‐1 and SHIP‐3 were administered the MMSE to assess cognitive status (Folstein et al., 1983).

In SHIP‐LEGEND as well as in SHIP‐3, an abridged version of the VLMT was used to assess short‐term learning as well as delayed retrieval. Subjects were asked to remember semantically unrelated nouns from an orally presented list in three consecutive encoding runs. Participants had 120 s after each encoding run for immediate retrieval. A sum score of the correctly remembered terms of the three encoding runs was formed to measure short‐term learning. Late retrieval was tested 20 min after the first encoding trial. The number of correctly remembered words reflected long‐term retrieval. The English Auditory Verbal Learning Test showed satisfactory recall reliability (0.70) and a moderate test–retest reliability (0.55). A close correlation with the California Test of Verbal Learning (0.5–0.65) support its validity (Schmidt, & Lezak,^1996^). For the German VLMT, no metrics on reliability are available. However, results showed a high convergent validity with the AVLT (Helmstaedter, Lendt, & Lux, 2001).

Cognitive status of participants of age 60 and older in SHIP‐1 and SHIP‐3 was assessed using the MMSE. The MMSE includes simple questions and tasks in a number of areas: orientation, short‐time memory tasks (repeating three words with delay), arithmetic tasks, language use and comprehension, and basic executive and motor skills. Scores above 24 (out of 30) are considered as normal. Scores of 20–24 indicate mild dementia; 10–19 indicates moderate dementia; and below 10, a severe dementia is suspected (Grabe et al., 2009).

Childhood trauma was assessed using the 34‐item version of the CTQ, a widely used self‐report scale (Bernstein & Fink, 1998; Wingenfeld et al., 2010). It comprises five different subscales: emotional abuse, physical abuse, sexual abuse, emotional neglect, and physical neglect. Responses are made on a 5‐point Likert‐type scale to express the frequency of occurrence and ranges from “never true” to “very often true.” In addition to dimensional scoring procedures the manual provides threshold scores to determine the severity of abuse and neglect dimensions (none = 0, mild = 1, moderate = 2, and severe to extreme = 3). A subject was rated as positive for a subscale when a severity score of ≥2 (at least moderate) was reported. Factor structure and construct validity of the German version showed sufficient psychometric properties with some limitations due to the high inter‐correlations of the different subscales and a weak internal consistency of the physical neglect subscale (Klinitzke, Romppel, Häuser, Brähler, & Glaesmer, 2012). In our main analyses, we used the CTQ sum‐score based on all five subscales.

In SHIP‐LEGEND, the lifetime diagnosis of MDD according to DSM‐IV criteria was determined using the standardized and computerized Munich‐Composite International Diagnostic Interview (M‐CIDI) (Wittchen, Lachner, Wunderlich, & Pfister, 1998). The M‐CIDI is a fully structured interview‐based instrument for the assessment of eight major classes of DSM‐IV diagnoses including MDD over the lifespan. It was developed based on the WHO‐CIDI in order to enhance the feasibility while maintaining the reliability (Wittchen et al., 1998). Excellent psychometric properties for the M‐CIDI have been shown (Wittchen, 1994). The computer‐assisted interview was conducted by clinically experienced interviewers (psychologists). We used the lifetime diagnosis of depression as a dichotomous measure. In SHIP‐1 the screening questions of the Composite International Diagnostic Screener for mental disorders (CID‐S) was applied for the assessment of coexisting depressive symptoms (Wittchen, 1994). Depression was assessed by two questions covering the dimensions “depressed mood” and “loss of interest.” Participants in SHIP‐1 were given the lifetime diagnosis of depression if at least one positive answer was given (Terock et al., 2020).

### 2.3 Genetic data

The SHIP sample was genotyped using the Affymetrix Human SNP Array 6.0. The overall genotyping efficiency was 98.55%. Imputation of genotypes was performed using the HRCv1.1 reference panel and the Eagle and minimac3 software implemented in the Michigan Imputation Server for prephasing and imputation, respectively. SNPs (SNPs) with a Hardy–Weinberg‐Equilibrium *p*‐value < .0001, a call rate < 0.95, and a minor allele frequency (MAF) <1% were removed before imputation. Both SNPs, rs5522 (MAF 12%) and rs2070951 (MAF 50%), were directly genotyped with a call rate of 1 and 0.97, respectively.

The 5′ region haplotype of the MR‐gene (CA/GA/CG) was based on these two SNPs as described in ter Heegde et al. (2015). Haplotype frequencies in SHIP were similar to those found in other cohorts (Vinkers et al., 2015). To investigate the contribution of specific combinations of haplotypes, diplotypes (the specific combination of haplotypes) were calculated for each individual. As we have no clear allocation for the SNPs on both homolog chromosomes, the haplotypes for those subjects heterozygous for both SNPs is not deterministic. As the Haplotype combination GG hardly exists, all of these subjects were set to the remaining possible haplotype (see Table S1 for haplotype allocation). The three common MR haplotypes were coded 0, 1, or 2 and modeled as a linear effect (additive genetic model). MR diplotypes (the combination of the two haplotypes per individual on both chromosomes) were constructed by combinations of the three haplotypes and analyzed relative to the diplotype group (GA–GA) using dummy coding because GA haplotype shows a significant lower protein expression compared to the CA and CG (ter Heegde et al., 2015).

### 2.4 Statistical analyses

Subject characteristics were assessed by mean and standard deviations for metric variables and with numbers and percentages for categorical data. Sample comparisons were performed using *t* test for metric variables and chi‐squared test for categorical variables.

#### 2.4.1 Longitudinal association analyses

A regression model with robust standard errors was fitted assessing the longitudinal effect of the two SNPs, as well as the haplotypes and diplotypes on VLMT (immediate and delayed recall). The VLMT difference between SHIP‐3 and SHIP‐LEGEND was used as outcome adjusted for education, age, sex, CTQ score, and follow‐up time (fut) between SHIP‐LEGENDE and SHIP‐3. In parallel, the same model was additionally fitted for the VLMT baseline value from SHIP‐LEGEND as suggested by previous studies (Glymour, Weuve, Berkman, Kawachi, & Robins, 2005). An association had to be significant in both models to be considered as valid. A similar model was used to assess the longitudinal effect of the SNPs and haplotype on MMSE between SHIP‐1 and SHIP‐3 (see equations below).

$${\text{VLMT}}_{\text{difference}}=\text{SNP}+\text{age}+\text{sex}+\text{education}+{\text{fut}}_{\text{VLMT}}+\text{CTQ}\left(+{\text{VLMT}}_{\text{SHIP}-\text{LEGEND}}\right)$$

$${\text{MMSE}}_{\text{difference}}=\text{SNP}+\text{age}+\text{sex}+\text{education}+{\text{fut}}_{\text{MMSE}}+\text{CTQ}\left(+{\text{MMSE}}_{\text{SHIP}-1}\right)$$

For both outcomes, a linear regression model was used. Histograms for the outcome variables of VLMT and MMSE in SHIP‐3 can be found in the supplement (Figures S1 and S2). In all analyses, age was treated nonlinear as restricted cubic splines with four knots based on Harrell Jr. (2015). The SNPs were categorized as 0/1 variable to ease interpretation of effects (rs5522 AA vs. AG/GG; rs2070951 GG vs. GC/CC).

In sensitivity analyses, these models were also adjusted for the effect of lifetime depression. Additionally, we examined the interaction effect between genetic variants and haplotypes with CTQ score on VLMT, as well as MMSE score difference.

As we analyzed the effects of two SNPs on two measures of cognition for VLMT (immediate/delayed recall), we set the Bonferroni corrected p‐value threshold *p*_corrected_ < .0125 correcting for *N* = 4 tests. For the MMSE analyses, we corrected for the effect of two SNPs (*p* < .025).

In addition, we performed exploratory sex‐stratified analyses to account to potential sex‐specific effects (see Tables S6–S9).

Analyses were performed using STATA/MP software, version 14 (StataCorp LP).

## 3 RESULTS

A sample characteristic for the two different analysis models and the different sample waves is given in Table 1. Analyses were performed only on complete cases for baseline and follow‐up. Thus, Table S2 compares baseline values for individuals with and without follow‐up data. As expected, those participants that were able to participate in the follow‐up SHIP‐3 were significantly younger, better educated, and showed higher VLMT scores at baseline than those participants without follow‐up.

**Table 1:** Sample characteristic for the different sample waves analyzed for VLMT and MMSE

Variable	SHIP‐LEGEND	SHIP‐3	Comparison sample waves for VLMT (*N* = 1,318)
Age (mean [*SD*])	53.8 (12.6)	60.1 (12.6)	
Sex			
Males	591 (45%)		
Females	727 (55%)		
Education			
<10 years	272 (21%)		
=10 years	757 (57%)		
>10 years	289 (22%)		
Follow‐up time in years (mean [*SD*])	6.4 (0.7)		
CTQ score (mean [*SD*])	33.7 (9.8)		
Lifetime depression	227 (17%)	NA	
rs5522			
AA	1,020 (77%)		
AG	280 (21%)		
GG	18 (2%)		
rs2070951			
GG	324 (24%)		
GC	667 (51%)		
CC	327 (25%)		
Haplotype CA (38.1%)			
0	505 (38.3%)		
1	621 (47.1%)		
2	192 (14.6%)		
Halotype GA (49.9%)			
0	327 (24.8%)		
1	667 (50.6%)		
2	324 (24.6%)		
Haplotype CG (12.0%)			
0	1,020 (77.4%)		
1	280 (21.4%)		
2	18 (1.4%)		
Haplotype GG (0%)			
0	1,318 (100%)		
1	0 (0%)		
2	0 (0%)		
Diplotypes			
GA/CA	504 (38.2%)		
GA/GA	324 (24.6%)		
CA/CA	192 (14.6%)		
CG/GA	163 (12.3%)		
CG/CA	117 (8.9%)		
CG/CG	18 (1.4%)		
CA/GG	0 (0%)		
VLMT immediate recall (meand [*SD*])	25.6 (5.8)	25.8 (6.5)	*T* = −1.8 *p* = .07
VLMT delayed recall (mean [*SD*])	8.3 (3.0)	8.3 (3.4)	*T* = −0.1 *p* = .92

Variable	SHIP‐1	SHIP‐3	Comparison sample waves foR MMSE (*N* = 377)
Age (mean [*SD*])	65.9 (4.9)		
Sex			
Males	188 (50%)		
Females	189 (50%)		
Education			
<10 years	205 (54%)		
=10 years	96 (26%)		
>10 years	76 (20%)		
Follow‐up time in years (mean [*SD*])	10.7 (0.6)		
CTQ score (mean [*SD*])	33.9 (8.5)		
Lifetime depression	72 (19%)	NA	
rs5522			
AA	277 (74%)		
AG	96 (25%)		
GG	4 (1%)		
rs2070951			
GG	85 (22%)		
GC	188 (50%)		
CC	104 (28%)		
Haplotype CA (38.7%)			
0	141 (37.4%)		
1	180 (47.7%)		
2	56 (14.9%)		
Haplotype GA (47.5%)			
0	104 (27.5%)		
1	188 (49.9%)		
2	85 (22.5%)		
Haplotype CG (13.8%)			
0	277 (73.4%)		
1	96 (25.5%)		
2	4 (1.1%)		
Haplotype GG (0%)			
0	377 (100%)		
1	0 (0%)		
2	0 (0%)		
Diplotypes			
GA/CA	136 (36.1%)		
GA/GA	85 (22.5%)		
CA/CA	56 (14.9%)		
CG/GA	52 (13.8%)		
CG/CA	44 (11.6%)		
CG/CG	4 (1.1%)		
CA/GG	0 (0%)		
MMSE score (mean [*SD*])	28.4 (1.6)	27.8 (2.0)	*T* = −5.6 *p* < .001

Abbreviations: CTQ, childhood trauma questionnaire; MMSE, Mini‐Mental Status Examination; *SD*, standard deviation; SHIP, Study of Health in Pomerania; VLMT, Verbal Learning and Memory Test.

### 3.1 Longitudinal analysis on VLMT

**Figure 1:**
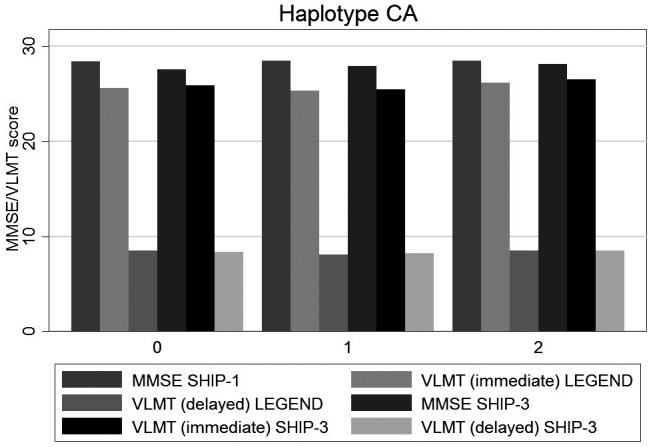
Associations of CA haplotype with VLMT/MMSE scores. The x‐axis shows the number of the haplotype and the y‐axis VLMT/MMSE scores. VLMT, Verbal Learning and Memory Test

**Figure 2:**
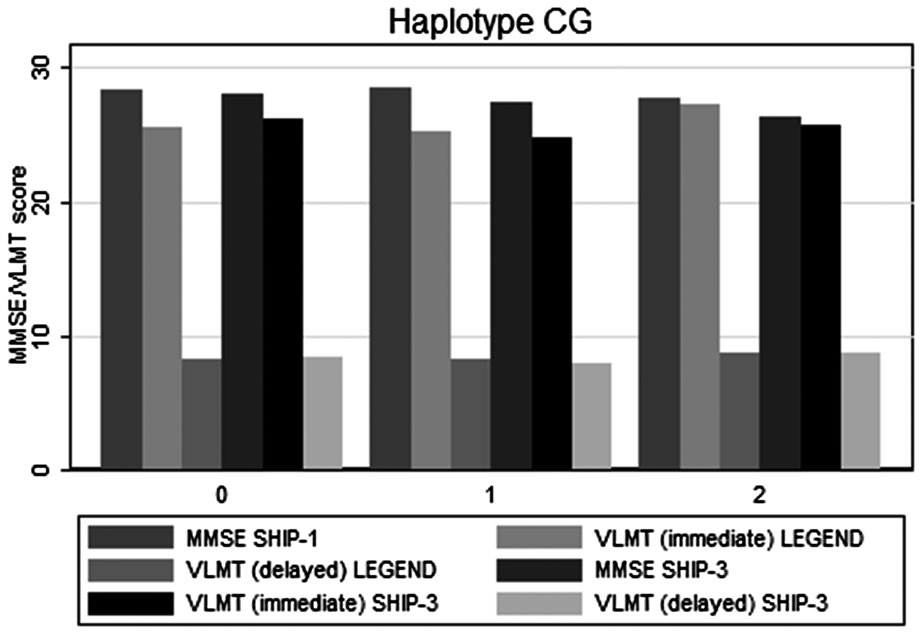
Associations of CG haplotype with VLMT/MMSE scores. The x‐axis shows the number of the haplotype and the y‐axis VLMT/MMSE scores. VLMT, Verbal Learning and Memory Test

**Figure 3:**
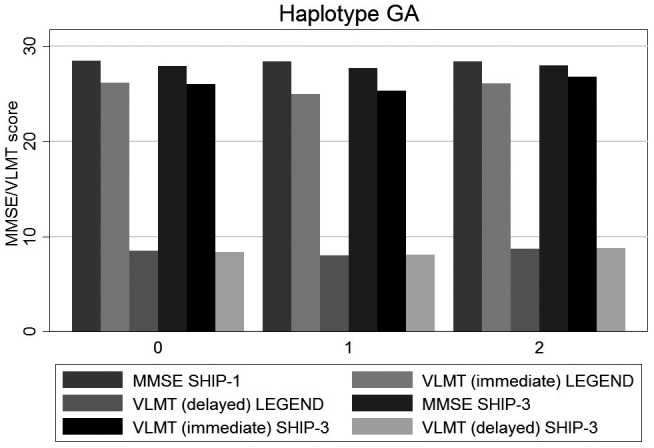
Associations of GA haplotype with VLMT/MMSE scores. The x‐axis shows the number of the haplotype and the y‐axis VLMT/MMSE scores. VLMT, Verbal Learning and Memory Test

The analyses on VLMT difference between SHIP‐LEGEND and SHIP‐3 were performed to assess the longitudinal effect of rs5522 and rs2070951 and their haplo/diplotypes. Detailed results are given in Table 2 and Figures 1, 2, 3.

**Table 2:** Results for the association analyses on VLMT differences between SHIP‐LEGEND and SHIP‐3. Beta estimates, 95% confidence intervals, and p‐values

Predictor^a^	VLMT immediate recall (*N* = 1,318)		VLMT delayed recall (*N* = 1,318)	
	No additional adjustment for baseline VLMT	Additional adjustment for baseline VLMT	No additional adjustment for baseline VLMT	Additional adjustment for baseline VLMT
rs2070951 (CC/CG vs. GG)	*β* = 0.54	***β* = 0.75**	*β* = 0.01	*β* = 0.22
	CI = [−0.12, 1.20]	**CI = [0.18, 1.31]**	CI = [−0.33, 0.35]	CI = [−0.08, 0.52]
	*p* = .11	***p* = 9.8E−3**	*p* = .94	*p* = .15
rs5522 (GG/GA vs. AA)	***β* = 0.96**	***β* = 1.00**	*β = 0.38*	*β = 0.40*
	**CI = [0.34, 1.57]**	**CI = [0.48, 1.52]**	*CI = [0.03, 0.74]*	*CI = [0.08, 0.71]*
	***p* = 2.3E−3**	***p* = 1.8E−4**	*p = .035*	*p = .014*
Haplotype CA	*β* = −0.04	*β* = 0.03	*β* = 0.04	*β* = 0.01
	CI = [−0.44, 0.37]	CI = [−0.32, 0.37]	CI = [−0.17, 0.25]	CI = [−0.17, 0.19]
	*p* = .86	*p* = .88	*p* = .68	*p* = .91
Haplotype GA	*β* = 0.44	*β* = 0.38	*β* = 0.10	*β* = 0.14
	CI = [0.03, 0.84]	CI = [0.04, 0.73]	CI = [−0.10, 0.31]	CI = [−0.04, 0.32]
	*p* = .034	*p* = .028	*p* = .33	*p* = .13
Haplotype CG	***β* = −0.95**	***β* = −0.96**	*β* = *−0.34*	*β* = *−0.35*
	**CI = [−1.52, −0.38]**	**CI = [−1.44, −0.49]**	*CI = [−0.66, −0.02]*	*CI = [−0.64, −0.07]*
	***p* = 1.2E−3**	***p* = 6.9E−5**	*p = .04*	*p = .015*
Diplotypes (ref. GA/GA)				
GA/CA	*β* = −0.24	*β* = −0.56	*β* = 0.15	*β* = −0.12
	CI = [−0.96, 0.49]	CI = [−1.19, 0.06]	CI = [−0.23, 0.53]	CI = [−0.46, 0.21]
	*p* = .52	*p* = .076	*p* = .43	*p* = .47
CA/CA	*β* = −0.38	*β* = −0.30	*β* = 0.01	*β* = −0.08
	CI = [−1.32, 0.57]	CI = [−1.10, 0.50]	CI = [−0.46, 0.49]	CI = [−0.49, 0.33]
	*p* = .43	*p* = .46	*p* = .96	*p* = .71
CG/GA	*β* = −0.78	***β* = −1.26**	*β* = −0.14	*β* = −0.39
	CI = [−1.68, 0.11]	**CI = [−2.03, −0.49]**	CI = [−0.66, 0.38]	CI = [−0.84, 0.05]
	*p* = .086	***p* = 1.4E−3**	*p* = .59	*p* = .083
CG/CA	***β* = −1.41**	***β* = −1.26**	*β* = −0.54	*β* = *−0.58*
	**CI = [−2.46, −0.35]**	**CI = [−2.16, −0.36]**	CI = [−1.12, 0.05]	*CI = [−1.12, −0.03]*
	***p* = 9.1E−3**	***p* = 6.1E−3**	*p* = .071	*p = .037*
CG/CG	*β* = *−2.71*	***β* = −2.53**	*β* = −0.29	*β* = −0.50
	*CI = [−5.18, −0.24]*	**CI = [−4.38, −0.69]**	CI = [−1.52, 0.93]	CI = [−1.54, 0.54]
	*p = .032*	***p* = 7.1E−3**	*p* = .64	*p* = .35

Abbreviations: CTQ, childhood trauma questionnaire; SHIP, Study of Health in Pomerania; VLMT, Verbal Learning and Memory Test.

^a^ Analyses adjusted for age (nonlinear), sex, education, CTQ score, follow‐up time (and VLMT score at baseline SHIP‐LEGEND); results significant after multiple testing correction (*p* < .0125) are highlighted in bold and nominal significant results (*p* < .05) are highlighted in italic.

The AA genotype of rs5522 was significantly associated with a higher VLMT score difference between SHIP‐3 and SHIP‐LEGEND for immediate recall (*β* = 0.96, *p* = 2.3E‐3) but for delayed recall (*β* = 0.38, *p* = .035) only on a nominal significant level. In the haplotype analysis, a significant effect was only found for the CG haplotype associated with lower immediate recall score difference (*β* = −0.95, *p* = 1.2E‐3). For delayed recall, this effect was only nominal significant (*β* = −0.34, *p* = .04). The GA haplotype revealed a nominal significant effect on VLMT immediate recall score difference (*β* = 0.44, *p* = .034). In diplotype analyses, the diplotypes GC/CA and CG/CG revealed significantly lower VLMT score differences for immediate recall when compared to GA/GA as reference (see Table 2). Additional adjustment for MDD lifetime did not affect the significance of the results (data not shown).

### 3.2 Longitudinal analysis on MMSE

The analyses on MMSE differences between SHIP‐1 and SHIP‐3 revealed similar results than those for VLMT immediate recall (see Table 3 and Figures 1, 2, 3).

**Table 3:** Results for the association analyses on MMSE differences between SHIP‐1 and SHIP‐3. Beta estimates, 95% confidence intervals, and p‐values

Predictor^a^	MMSE score difference (*N* = 377)	
	No additional adjustment for baseline MMSE	Additional adjustment for baseline MMSE
rs2070951 (CC/CG vs. GG)	*β* = 0.34, CI=[−0.17, 0.85], *p* = .18	*β* = 0.31, CI = [−0.10, 0.72], *p* = .14
rs5522 (GG/GA vs. AA)	***β* = 0.62, CI = [0.09, 1.15], *p* = .021**	***β* = 0.59, CI = [0.10, 1.08], *p* = .019**
Haplotype CA	*β* = 0.17, CI = [−0.13, 0.47], *p* = .28	*β* = 0.17, CI = [−0.09, 0.43], *p* = .21
Haplotype GA	*β* = 0.12, CI = [−0.17, 0.41], *p* = .43	*β* = 0.11, CI = [−0.14, 0.36], *p* = .38
Haplotype CG	***β* = −0.61, CI = [−1.07, −0.14], *p* = .011**	***β* = −0.60, CI = [−1.04, −0.16], *p* = 8.2E−3**
Diplotypes (ref. GA/GA)		
GA/CA	*β* = −0.18, CI = [−0.77, 0.41], *p* = .55	*β* = −0.14, CI = [−0.61, 0.32], *p* = .55
CA/CA	*β* = −0.05, CI = [−0.69, 0.59], *p* = .88	*β* = −0.06, CI = [−0.58, 0.47], *p* = .83
CG/GA	***β* = −0.93, CI = [−1.74, −0.12], *p* = .025**	***β* = −0.88, CI = [−1.61, −0.15], *p* = .019**
CG/CA	*β* = −0.41, CI = [−1.23, 0.42], *p* = .33	*β* = −0.32, CI = [−1.06, 0.42], *p* = .40
CG/CG	***β* = −1.49, CI = [−2.08, −0.90], *p* = 9.8E‐7**	***β* = −1.85, CI = [−2.92, −0.79], *p* = 6.7E‐4**

Abbreviations: CTQ, childhood trauma questionnaire; SHIP, Study of Health in Pomerania.

^a^ Analyses adjusted for age (nonlinear), sex, education, CTQ score, follow‐up time (and MMSE score at baseline SHIP‐1); results significant after multiple testing correction (MMSE: *p* < .025) are highlighted in bold and nominal significant results (*p* < .05) are highlighted in italic.

The AA genotype of rs5522 was significantly associated with a higher MMSE score difference between SHIP‐3 and SHIP‐1 (*β* = 0.62, *p* = .021). In the haplotype analysis, a significant effect was only found for the CG haplotype associated with lower MMSE score difference (*β* = −0.61, *p* = .011). The other two haplotypes revealed no effect on MMSE score difference. Diplotype analyses showed a significantly lower MMSE score difference for CG/GA and CG/CG compared to GA/GA as reference (see Table 3). Additional adjustment for MDD lifetime did not affect the significance of the results (data not shown).

### 3.3 Interactions with CTQ score

For the interaction between CTQ score and SNPs/haplotype on VLMT and MMSE score differences, there was no significant association, neither for the individual SNPs nor for the three common haplotypes (see Tables S3 and S4). The direct effect of CTQ score and its subscores on VLMT and MMSE score differences are given in the supplement (Table S5).

### 3.4 Sex‐stratified analyses

Results of the sex‐stratified analyses are shown in the Tables S6–S9. In male subjects, we found the association of the rs5522 polymorphism (*β* = 1.24; *p* = .002) as well as of the CG haplotype were maintained (*β* = −1.17; *p* = .001), while in all other analyses, significance was lost. While this loss of significance may be due to the reduced statistical power in the sex‐separated analyses, beta estimates still indicated relevant associations of the SNPs and haplotypes with the memory outcomes particularly in male subjects.

## 4 DISCUSSION

Considering the previously shown role of stress and HPA‐axis dysregulation on cognition and memory loss (ter Heegde et al., 2015), we sought to investigate the role of functional polymorphisms of the mineralocorticoid receptor gene (*NR3C2*) on cognitive decline in a general population study and also in interaction with childhood trauma. We found that both polymorphisms were significantly associated with immediate recall of words and, in the subgroup of older subjects, with global cognitive functioning. Analyzing the functional MR haplotypes, our results revealed that haplotype 3 (CG) of MR was associated with increased loss of memory performance and global cognitive functioning as compared to the reference haplotype 1. This result was supported by diplotype analyses, where diplotypes containing the CG haplotype showed increased memory decline. Childhood trauma showed no main effects on cognitive functioning in SHIP‐3 and only a weak interaction effect with the functional haplotypes on MMST, but not on verbal declarative memory.

In detail, we found that the GG genotype of rs2070951 as well as the AA genotype of rs5522 are associated with diminished memory decline as compared to the other genotypes. These results appear contradictory considering previous studies on in vitro functionality of the two SNPs which suggested that the GG genotype of rs2070951 is associated with low gene expression and functionality while the AA genotype represents the high functionality genotype (Van Leeuwen et al., 2011). Moreover, haplotype 3 (CG), but not 2 (CA) was significantly associated with reduced memory and cognitive performance as compared to the reference haplotype (GA). Previous results demonstrated that haplotype 3 was linked to a slightly enhanced protein expression in comparison to haplotype 1, while haplotype 2 was linked to the highest expression and activity of the MR (Van Leeuwen et al., 2011). However, these results were obtained in response to pharmacological cortisol stimulation. In contrast, in vitro testing under basal, nonstimulated conditions revealed that haplotype 3 showed the lowest promoter activity (Klok et al., 2011; Klok et al., 2011) of the three haplotypes.

Given that the participants of this study were not exposed to stress‐induced or pharmacological cortisol administration and as we did not find interactions between childhood trauma and the genetic polymorphisms, our findings suggest that haplotypes associated with reduced MR expression and activation under baseline conditions are linked with enhanced cognitive decline. Thereby, our results are in line with previous findings suggesting that enhanced MR functionality may mitigate the detrimental effects of chronic stress on cognitive performance and memory‐relevant brain regions (ter Heegde et al., 2015). For example, activation of the intracellular genomic MR is associated with long‐term potentiation of hippocampal neurons (Kerr, Huggett, & Abraham, 1994) whereas increasing activation of GR due to elevated cortisol levels diminishes hippocampal long‐term potentiation and inhibits MR activation (Diamond, Bennett, Fleshner, & Rose, 1992). Lai et al. (2007) reported that MR overexpressing mice showed attenuated neuronal loss and enhanced memory following transient cerebral ischemia. In contrast, transgenic mice with inactivated limbic MR receptors showed impaired learning, deficient working memory, as well as reduced density and neurogenesis of hippocampal granule cells (Berger et al., 2006). Moreover, in vitro experimental results indicated that the MR moderates GR‐mediated hippocampal cell death such that MR activation counteracted corticosteroid stimulated apoptosis, while MR antagonism caused an increase in GR‐mediated hippocampal apoptosis (Crochemore et al., 2005). Still, in addition to these slowly acting genomic steroid actions, increasing evidence suggests that fast acting nongenomic steroid effects mediated by membrane bound MR and GR are critical to the stress‐coping capacities as well as to stress‐related memory formation and retrieval (Joels et al., 2008; Joëls, Sarabdjitsingh, & Karst, 2012). Given these complementary and interactive effects of the brain steroid receptors on neuronal structure and function, future studies addressing the putative main and interactive effects of MR and GR gene polymorphisms are needed. Finding no significant interactions between childhood maltreatment and the two SNPs on memory is in contrast to various earlier findings showing such effects on different neuroendocrine, brain structural, and psychiatric outcomes (Baes, de Carvalho Tofoli, Martins, & Juruena, 2012; Vinkers et al., 2015). Specifically, it has been suggested that the *NR3C2* moderates the effects of early life stress on reduced hippocampal and amygdala volumes (Gerritsen et al., 2017), brain regions with a high relevance for the functioning of the declarative memory. However, previous findings on the link between early life stress and MR expression and activity are not fully consistent and also point at a bidirectional relationship with results showing that stress induced MR expression in rodents (Gesing et al., 2001; Rybnikova, Glushchenko, Churilova, Pivina, & Samoilov, 2011). Also, psychopathologies may have influenced our results and may partly explain the differences to previous studies. While all results were adjusted for depression, different other psychopathologies which are typically highly associated with childhood trauma like substance abuse (Min, Farkas, Minnes, & Singer, 2007) or dissociation (Terock et al., 2016) may have contributed to the divergent finding.

Based on previous findings showing that the MR haplotype moderates the effects of estradiol and progesterone on emotional information processing (Hamstra, de Kloet, Quataert, Jansen, & Van der Does, 2017) and studies showing sex‐specific effects of the MR haplotype on HPA‐axis activity (Van Leeuwen et al., 2011), dispositional optimism (Klok et al., 2011; Klok et al., 2011), and depression (Vinkers et al., 2015), we performed additional sex‐stratified results. Due to the reduced statistical power, *p*‐values were generally weaker in all results. However, significance was maintained for the associations of rs5522 and the CG haplotype with immediate recall in the male subgroup, while in the female subgroup none of the performed analyses reach significance. Moreover, beta estimates stayed robust and suggest relevant associations of both polymorphisms and the corresponding haplotypes particularly in the male subgroup (Tables S6 and S7), indicating that sex‐specific effects of these polymorphisms on memory performance may exist. However, sex X SNP interaction analyses did not reveal any significant result in our sample.

The MR and limbic structures are not only target structures of glucocorticoids, but also are involved in the regulation of the HPA‐axis. Specifically, the intracellular genomic MR is considered to take in a regulatory role for the HPA‐axis as it mediates a proactive negative cortisol feedback, thereby diminishing HPA‐axis activity and determining the threshold for HPA‐axis activation (De Kloet, Vreugdenhil, Oitzl, & Joëls, 1998). In this context, it could be speculated that lower MR functionality leads to a diminished negative HPA‐axis feedback loop which subsequently results in elevated cortisol levels. Results from preceding studies consistently showed that enhanced HPA‐axis activation and cortisol levels are associated with poorer cognitive function and faster cognitive decline in subjects with and without dementia (Huang et al., 2009; Lupien et al., 1994). More specifically, given that the density of the MR in the brain is highest in limbic regions and particularly the hippocampus, these results may contribute to elucidate the mechanism underlying the associations of lasting elevations of glucocorticoid levels with reduced hippocampal volumes and hippocampal‐dependent memory in animals and humans (Fuchs et al., 2001; Lupien et al., 1998).

Regarding the declarative memory, we found that immediate recall was diminished in carriers of haplotype 3, whereas delayed retrieval of words was largely unaffected, indicating that particularly early steps of memory formation like encoding and attentive information processing are affected. There is converging evidence that memory performance depends on both, the cytoplasmic genomic MR as well as the fast acting, membrane‐bound MR. Karst et al. (2005) reported that hippocampal membrane‐bound MR are crucial for nongenomic glutamatergic signaling in response to stress and corticosteroids. Khaksari, Rashidy‐Pour, and Vafaei (2007) reported that the MR antagonist spironolactone, but not the protein synthesis inhibitor anisomycin was able to reverse corticosterone induced memory deficits in rats, indicating that memory deficits in response to corticosteroids are facilitated via nongenomic mechanisms. Likewise, Dorey et al. (2011) found memory impairments in response to a corticosterone conjugate which is unable to cross the cell‐membrane. These effects could not be blocked by anisomycin, but by a MR antagonist, suggesting that the membrane‐bound MR has direct effects on memory performance.

In addition to facilitating rapid, but reversible stress effects on memory formation, the membrane‐located MR‐receptor is also involved in the regulation of neural plasticity (Karst et al., 2005). Early memory formation highly depends on the integrity of the prefrontal cortex, which also shows a high density of both GR and MR (Diorio, Viau, & Meaney, 1993) and which was found to be particularly sensitive to the detrimental effects of chronic stress (Arnsten, 2009). Also, chronic psychosocial stress was found to alter expression and distribution of GR and MR in primate hippocampus and prefrontal cortex (Patel, Katz, Karssen, & Lyons, 2008) and to be associated with early, but not delayed recall (Terock et al., 2018) in humans. Still, considering the result of reduced MMSE scores in haplotype 3 carriers suggests that, at least in older individuals, faster decline involves various domains of cognitive functioning.

In general, we found a decline in cognitive functioning in the complete analytic sample as reflected by the MMST, while immediate recall showed only a weak, nonsignificant (*p* = .07) decline and delayed recall remained largely unchanged. These effects may be due to the significantly older subsample with data on MMST as well as with the shorter time‐span observation period for data on the VLMT.

Given that we did not find interactive effects of childhood trauma with *NR3C2* haplotypes, our results indicate that enhanced MR functionality increases the risk for faster cognitive decline even under normal cortisol levels. Moreover, in contrast to previous results showing an association between stress, early life adversity and cognitive dysfunction (Gould et al., 2012; Terock et al., 2018, 2019), we did not observe an effect of childhood trauma on cognitive decline in our subsamples of longitudinal memory information. Still, in most previous studies, cross‐sectional data on associations of childhood trauma with cognitive functioning were assessed, while in this study a longitudinal approach was applied. It is well conceivable that childhood trauma is associated with impaired cognitive functioning at both time points, but not with increased cognitive decline. Therefore, our findings do not necessarily contradict the results from previous cross‐sectional studies.

Some limitations of our study need to be acknowledged: We concentrated on the most widely investigated genetic polymorphisms of the MR receptor gene (rs5522 and rs2070951) and the combined haplotypes in order to keep the number of performed analyses small. However, other functional polymorphisms of the *NR3C2*, particularly rs5520 and rs17581262, may interact with the SNPs tested in this study or may show main effects as indicated by previous results (Gerritsen et al., 2017). Moreover, the SNPs investigated in this study were assumed to reflect the functionality of the MR. However, as noted above, there is some inconsistency in studies on the activity and expression associated with the haplotypes, perhaps due to stimulated versus nonstimulated conditions (DeRijk et al., 2008; Van Leeuwen et al., 2011). As we did not have data on the epigenetic effects of theses polymorphisms, we are not able to finally determine whether our results reflect increased or decreased MR functionality. Also, while we used childhood trauma as a proxy for HPA‐axis dysregulation, previous studies on the neuroendocrine effects of this stress factor revealed a large heterogeneity in findings with some studies showing a hypoactivated (Carpenter et al., 2007; Elzinga et al., 2008; Suzuki, Poon, Papadopoulos, Kumari, & Cleare, 2014) and other studies showing a hyperactivated (Goldman‐Mellor et al., 2012; Heim et al., 2002; Rinne et al., 2002) HPA‐axis. Therefore, finding no main or interactive effects of childhood trauma with MR haplotypes on cognitive decline does not allow to draw causal conclusions on putative moderating effects of cortisol levels and the MR. Studies investigating the relation of MR gene polymorphisms and cognitive decline should take other chronic stress factors as well as cortisol levels into account. A strength of our study is the large and well‐characterized general population sample. However, in candidate gene studies, a replication by an independent sample is preferable. While unfortunately our dataset does not comprise another independent cohort with longitudinal data, our approach of analyzing the MMSE in a subgroup of our sample may be regarded as an internal validation of our result. Finally, our result does not match with findings from genome‐wide association studies on education/memory/intelligence (GWAS) (Barral et al., 2012; Smith et al., 2018). Specifically, one large GWAS meta‐analysis with contribution from data of the SHIP study identified one single SNP, rs4420639, to be associated with poorer delayed recall (Debette et al., 2015). Additional SNPs were found to be associated with specific tests in subsets of the sample. However, these results were based on cross‐sectional data and restricted to individuals aged 45 or older, which limits the comparability.

In all, our findings support concept that the MR takes in a key role in cognitive functioning and particularly memory performance and provide additional evidence that reduced MR expression and activation represents a risk factor for accelerated cognitive decline irrespective of the effects of early life stress. In the light of earlier studies, these effects may be not only due to a reduced regulating effect on the HPA‐axis (ter Heegde et al., 2015), but also to diminished functioning of membrane‐located fast‐acting MR. In this context, our results suggest that reduced MR functionality contributes to transfer the detrimental effects of elevated cortisol levels on memory‐relevant brain structures like the hippocampus and the prefrontal cortex.

Future studies on the effects of the MR gene, MR expression, and brain volumes are needed in order clarify whether the functional cognitive decline is reflected by brain structural alterations. Also, with respect to findings pointing at a modulating effect of MR activity on GR‐mediated hippocampal neuronal apoptosis, the question whether these receptors and their corresponding genes interact in their putative effects on cognitive declines represents an interesting field which is worth to be addressed in future studies.

## CONFLICTS OF INTEREST

All authors declare no competing interests.

## AUTHOR CONTRIBUTION

J. T. and S. V. conceived of the presented idea; S. V. performed computations with support and verification of K. W. and A. T.; J. T. wrote the manuscript with input from D. J.; H. G. supervised the project; all authors discussed the results and the final version of the manuscript.

## Supporting information

Tables S1‐S9‐Figs S1‐S2

## Data Availability

The data that support the findings of this study are available on request from the corresponding author. The data are not publicly available due to privacy or ethical restrictions.
