# Supplementary material for: Functional polymorphisms of the mineralocorticoid receptor gene NR3C2 are associated with diminished memory decline: Results from a longitudinal general‐population study
Source: Mol Genet Genomic Med. 2020 Jun 18;8(9):e1345. doi: 10.1002/mgg3.1345 (PMC7507013; doi:10.1002/mgg3.1345)
Supplement: Supplementary file 1 — Tables S1‐S9‐Figs S1‐S2 [file MGG3-8-e1345-s001.docx]

**Supplementary Tables and Figures**

**Tables**

**Table S1.** Haplotype reconstruction in SHIP based on rs5522 and rs2070951

|  | **rs2070951_GG** | **rs2070951_GC** | **rs2070951_CC** |
| --- | --- | --- | --- |
| **rs5522_AA** | 2*GA | 1*GA/1*CA | 2*CA |
| **rs5522_AG** | NA | 1*GA/1*CG | 1*CA/1*CG |
| **rs5522_GG** | NA | NA | 2*CG |

*NA: combination not available in SHIP

**Table S2**

Comparison of SHIP-LEGEND participants with and without follow-up in SHIP-3.

|  | Sample with follow-up (1318) | Sample without follow-up (746) | comparison |
| --- | --- | --- | --- |
| Age | 53.8 (12.6) | 58.4 (15.2) | T=7.5, p<0.001 |
| sex (Males) | 591 (45%) | 382 (51%) | Chi2=7.7, p=0.006 |
| education  <10 years  10 years  >10 years | 272 (21%)  757 (57%)  289 (22%) | 285 (38%)  339 (45%)  122 (17%) | Chi=74.3, p<0.001 |
| CTQ sumscore | 33.7 (9.8) | 34.0 (8.7) | T=0.83, p=0.41 |
| Baseline VLMT (immediate recall) | 25.6 (5.8) | 22.9 (6.7) | T=-9.6, p<0.001 |
| Baseline VLMT (delayed recall) | 8.3 (3.0) | 7.3 (3.3) | T=-7.4, p<0.001 |
| rs5522 | 1020 (77%)  280 (21%)  18 (2%) | 589 (79%)  146 (20%)  11 (1%) | P=0.64 |
| rs2070951 | 324 (24%)  667 (51%)  327 (25%) | 202 (27%)  369 (50%)  175 (23%) | P=0.44 |

**Table S3**

Results for the interaction analyses (SNPs/haplotypes*CTQ sum score) on VLMT differences between SHIP-LEGEND and SHIP-3. Beta estimates, 95% confidence intervals and p-values.

| Predictor (CTQ*) | VLMT immediate recall (N=1318) | | VLMT delayed recall (N=1318) | |
| --- | --- | --- | --- | --- |
|  | **No additional adjustment for baseline VLMT** | **Additional adjustment for baseline VLMT** | **No additional adjustment for baseline VLMT** | **Additional adjustment for baseline VLMT** |
| rs2070951 (CC/CG vs. GG) | β=0.01  CI=[-0.06, 0.08]  p=0.78 | β= -0.01  CI=[-0.08, 0.05]  p=0.76 | β=-0.02  CI=[-0.06, 0.03]  p=0.44 | β=-0.02  CI=[-0.06, 0.02]  p=0.27 |
| rs5522 (GG/GA vs. AA) | β=-0.01  CI=[-0.06, 0.05]  p=0.80 | β=-0.03  CI=[-0.09, 0.03]  p=0.32 | β=-0.01  CI=[-0.04, 0.02]  p=0.54 | β=-0.01  CI=[-0.04, 0.02]  p=0.33 |
| Haplotype CA | β=-0.01  CI=[-0.05, 0.03]  p=0.73 | β=-0.01  CI=[-0.04, 0.03]  p=0.74 | β=0.003  CI=[-0.02, 0.03]  p=0.80 | β=0.01  CI=[-0.02, 0.03]  p=0.60 |
| Haplotype GA | β=0.002  CI=[-0.04, 0.05]  p=0.94 | β=-0.01  CI=[-0.05, 0.03]  p=0.68 | β=-0.01  CI=[-0.04, 0.02]  p=0.51 | β=-0.01  CI=[-0.04, 0.01]  p=0.28 |
| Haplotype CG | β=0.01  CI=[-0.04, 0.06]  p=0.78 | β=0.03  CI=[-0.03, 0.08]  p=0.31 | β=0.01  CI=[-0.02, 0.04]  p=0.45 | β=0.02  CI=[-0.01, 0.04]  p=0.29 |

*analyses adjusted for age (non-linear), sex, education, follow-up time (and VLMT score at baselineSHIP-LEGEND); results significant after multiple testing correction (p<0.0125) are highlighted in bold, nominal significant results (p<0.05) are highlighted in italic.

**Table S4**

Results for the interaction analyses (SNPs/haplotypes*CTQ sum score) on MMSE differences between SHIP-1 and SHIP-3. Beta estimates, 95% confidence intervals and p-values.

| Predictor (CTQ*) | | MMSE score difference (N=377) | |
| --- | --- | --- | --- |
|  | **No additional adjustment for baseline MMSE** | | **Additional adjustment for baseline MMSE** |
| rs2070951 (CC/CG vs. GG) | β=-0.02, CI=[-0.08, 0.04], p=0.51 | | β=-0.02, CI=[-0.06, 0.03], p=0.43 |
| rs5522 (GG/GA vs. AA) | β=0.01, CI=[-0.04, 0.05], p=0.78 | | β=-0.002, CI=[-0.05, 0.04], p=0.92 |
| Haplotype CA | β=0.02, CI=[-0.01, 0.06], p=0.14 | | β=0.02, CI=[-0.01, 0.05], p=0.22 |
| Haplotype GA | β=-0.02, CI=[-0.06, 0.01], p=0.22 | | β=-0.02, CI=[-0.05, 0.01], p=0.16 |
| Haplotype CG | β=-0.005, CI=[-0.05, 0.04], p=0.83 | | β=0.004, CI=[-0.04, 0.04], p=0.83 |

*analyses adjusted for age (non-linear), sex, education, follow-up time (and MMSE score at baseline SHIP-1); results significant after multiple testing correction (MMSE: p<0.025) are highlighted in bold, nominal significant results (p<0.05) are highlighted in italic.

**Table S5**

Direct effects of CTQ sum score and the five sub-scores on VLMT and MMST score differences

| Predictor | VLMT immediate recall score difference | VLMT delayed recall score difference | MMSE score difference |
| --- | --- | --- | --- |
| CTQ sum score | β=0.00, p=0.99 | β=-0.002, p=0.79 | β=-0.02, p=0.97 |
| Emotional abuse | β=0.13, p=0.36 | β=-0.06, p=0.89 | β=-0.01, p=0.99 |
| Physical abuse | β=0.10, p=0.88 | β=-0.11, p=0.78 | β=-1.26, p=0.0084 |
| Sexual abuse | β=1.16, p=0.14 | β=-0.19, p=0.67 | β=-0.61, p=0.26 |
| Emotional neglect | β=0.34, p=0.44 | β=0.41, p=0.091 | β=0.34, p=0.44 |
| Physical neglect | β=0.10, p=0.88 | β=-0.11, p=0.78 | β=-1-26, p=0.0084 |

Analyses adjusted for age (non-linear), sex, education and follow-up time.

**Table S6**

Results for the association analyses on VLMT differences between SHIP-LEGEND and SHIP-3. Beta estimates, 95% confidence intervals and p-values. **Males only**.

| Predictor | VLMT immediate recall | | VLMT delayed recall | |
| --- | --- | --- | --- | --- |
|  | **No additional adjustment for baseline VLMT** | **Additional adjustment for baseline VLMT** | **No additional adjustment for baseline VLMT** | **Additional adjustment for baseline VLMT** |
| rs2070951 (CC/CG vs. GG) | β= 0.93  CI=[-0.1, 1.26]  p=0.075 | β= 1.06  *CI=[0.15, 1.97]*  *p=0.023* | β= 0.35  CI=[-0.19, 0.89]  p=0.21 | β=0.41  CI=[-0.08, 0.9]  p=0.1 |
| rs5522 (GG/GA vs. AA) | β= 1.17  CI=[0.27, 2.07]  p=0.11 | **β= 1.24**  **CI=[0.46, 2.02]**  **p=0.002** | β= 0.54  CI=[0.03, 1.06]  p=0.038 | β= 0.52  CI=[0.05, 0.98]  p=0.029 |
| Haplotype CA | β= -0.11  CI=[-0.7, 0.48]  p=0.73 | β= -0.08  CI=[-0.61, 0.45]  p=0.76 | β= -0.03  CI=[-0.35, 0.28]  p=0.85 | β= -0.02  CI=[-0.3, 0.26]  p=0.88 |
| Haplotype GA | β= 0.61  CI=[0, 1.21]  p=0.049 | β= 0.6  CI=[0.07, 1.13]  p=0.028 | β= 0.25  CI=[-0.09, 0.58]  p=0.15 | β= 0.21  CI=[-0.08, 0.51]  p=0.16 |
| Haplotype CG | **β= -1.13**  **CI=[-1.94, -0.33]**  **p=0.006** | **β= -1.17**  **CI=[-1.86, -0.49]**  **p=0.001** | β= -0.49  CI=[-0.94, 0.58]  p=0.031 | β= -0.44  CI=[-0.85, -0.02]  p=0.038 |

*analyses adjusted for age (non-linear), education, CTQ score, follow-up time (and MMSE score at baseline SHIP-1); results significant after multiple testing correction (MMSE: p<0.025) are highlighted in bold, nominal significant results (p<0.05) are highlighted in italic.

**Table S7**

Results for the association analyses on MMSE differences between SHIP-1 and SHIP-3. Beta estimates, 95% confidence intervals and p-values. **Males only**.

| Predictor | | MMSE score difference | |
| --- | --- | --- | --- |
|  | **No additional adjustment for baseline MMSE** | | **Additional adjustment for baseline MMSE** |
| rs2070951 (CC/CG vs. GG) | β=0.28, CI=[-0.41, 0.97], p=0.42 | | β=0.33, CI=[-0.18, 0.84], p=0.21 |
| rs5522 (GG/GA vs. AA) | β=0.68, CI=[-0.08, 1.44], p=0.077 | | β=0.58, CI=[-0.11, 1.27], p=0.096 |
| Haplotype CA | β=0.1, CI=[-0.28, 0.48], p=0.6 | | β=0.03, CI=[-0.3, 0.36], p=0.85 |
| Haplotype GA | β=0.22, CI=[-0.19, 0.63], p=0.3 | | β=0.26, CI=[-0.1, 0.62], p=0.16 |
| Haplotype CG | β=-0.64, CI=[-1.27, -0.01], p=0.046 | | β=-0.6, CI=[-1.18, -0.01], p=0.046 |

*analyses adjusted for age (non-linear), education, CTQ score, follow-up time (and MMSE score at baseline SHIP-1); results significant after multiple testing correction (MMSE: p<0.025) are highlighted in bold, nominal significant results (p<0.05) are highlighted in italic.

**Table S8**

Results for the association analyses on VLMT differences between SHIP-LEGEND and SHIP-3. Beta estimates, 95% confidence intervals and p-values. **Females only**.

| Predictor | VLMT immediate recall | | VLMT delayed recall | |
| --- | --- | --- | --- | --- |
|  | **No additional adjustment for baseline VLMT** | **Additional adjustment for baseline VLMT** | **No additional adjustment for baseline VLMT** | **Additional adjustment for baseline VLMT** |
| rs2070951 (CC/CG vs. GG) | β= 0.22  CI=[-0.64, 1.08]  p=0.62 | β=0.46  CI=[-0.26, 1.17]  p=0.21 | β=-0.23  CI=[-0.66, 0.19]  p=0.29 | β= 0.08  CI=[-0.29, 0.45]  p=0.68 |
| rs5522 (GG/GA vs. AA) | β= 0.72  CI=[-0.12, 1.56]  p=0.093 | β= 0.73  CI=[0.04, 1.43]  p=0.038 | β= 0.23  CI=[-0.26, 0.73]  p=0.36 | β= 0.27  CI=[-0.16, 0.7]  p=0.21 |
| Haplotype CA | β= 0.03  CI=[-0.52, 0.59]  p=0.91 | β= 0.16  CI=[-0.29, 0.61]  p=0.49 | β= 0.11  CI=[-0.17, 0.39]  p=0.44 | β= 0.04  CI=[-0.2, 0.29]  p=0.72 |
| Haplotype GA | β= 0.28  CI=[-0.26, 0.82]  p=0.3 | β= 0.17  CI=[-0.28, 0.61]  p=0.46 | β= -0.02  CI=[-0.27, 0.24]  p=0.9 | β= 0.07  CI=[-0.16, 0.3]  p=0.54 |
| Haplotype CG | β= -0.75  CI=[-1.56, 0.05]  p=0.066 | β= -0.74  CI=[-1.39, -0.09]  p=0.025 | β= -0.21  CI=[-0.66, 0.25]  p=0.38 | β= -0.27  CI=[-0.66, 0.12]  p=0.18 |

*analyses adjusted for age (non-linear), education, CTQ score, follow-up time (and VLMT score at baselineSHIP-LEGEND); results significant after multiple testing correction (p<0.0125) are highlighted in bold, nominal significant results (p<0.05) are highlighted in italic.

**Table S9**

Results for the association analyses on MMSE differences between SHIP-1 and SHIP-3. Beta estimates, 95% confidence intervals and p-values. **Females only**.

| Predictor | | MMSE score difference (N=377) | |
| --- | --- | --- | --- |
|  | **No additional adjustment for baseline MMSE** | | **Additional adjustment for baseline MMSE** |
| rs2070951 (CC/CG vs. GG) | β=0.33, CI=[-0.42, 1.09], p=0.39 | | β=0.21, CI=[-0.44, 0.87], p=0.52 |
| rs5522 (GG/GA vs. AA) | β=0.51, CI=[-0.23, 1.25], p=0.18 | | β=0.54, CI=[-0.16, 1.23], p=0.13 |
| Haplotype CA | β=0.21, CI=[-0.25, 0.67], p=0.36 | | β=0.3, CI=[-0.1, 0.69], p=0.15 |
| Haplotype GA | β=0.01, CI=[-0.41, 0.43], p=0.96 | | β=-0.06, CI=[-0.41, 0.29], p=0.74 |
| Haplotype CG | β=-0.51, CI=[-1.21, 0.18], p=0.15 | | β=-0.54, CI=[-1.19, 0.11], p=0.11 |

*analyses adjusted for age (non-linear), education, CTQ score, follow-up time (and MMSE score at baseline SHIP-1); results significant after multiple testing correction (MMSE: p<0.025) are highlighted in bold, nominal significant results (p<0.05) are highlighted in italic.

**Figures**

**Figure S1.** Histogram of all three outcome variables (score differences of VLMT immediate/delayed recall and MMSE score).


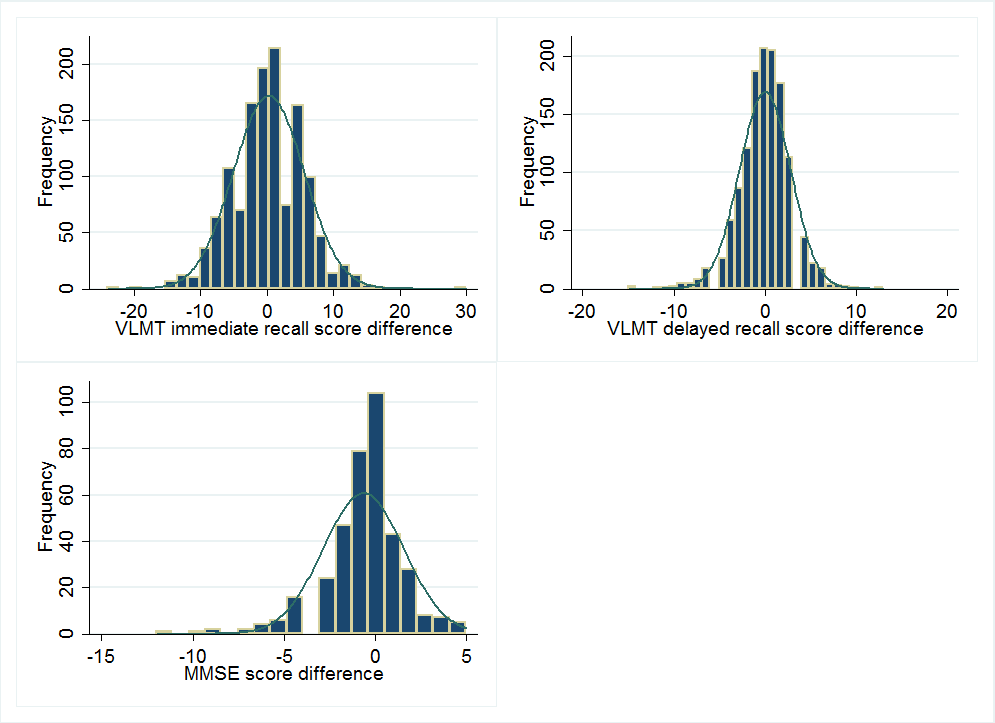


Figures


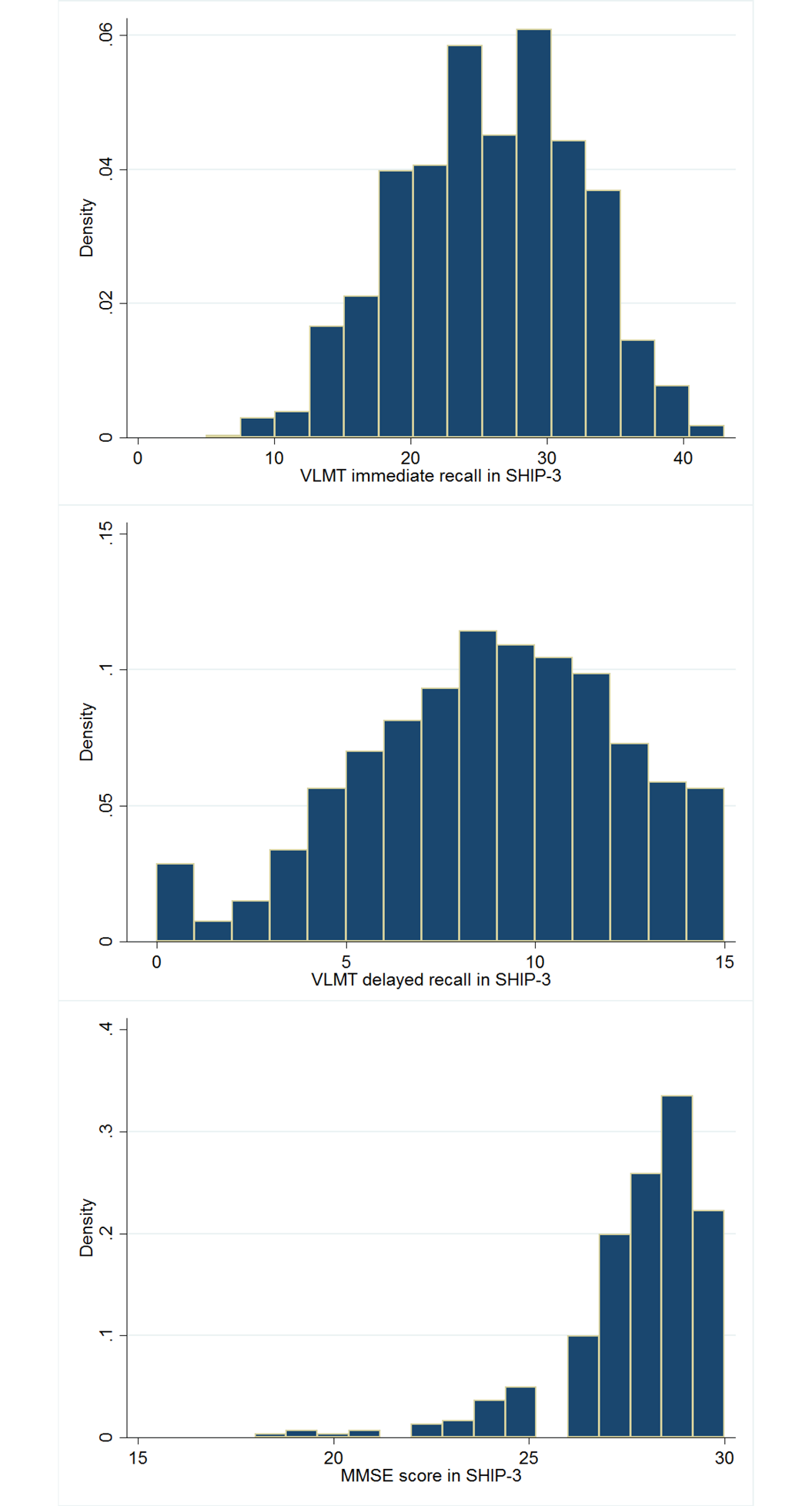


Figure S1. Histogram of all three outcome variables in SHIP-3 (VLMT immediate recall, VLMT delayed recall and MMSE score).
